# Numerical Simulation of Heat and Mass Transfer in Sludge Low-Temperature Drying Process

**DOI:** 10.3390/e24111682

**Published:** 2022-11-17

**Authors:** Zhenyu Wang, Qiang Wang, Ju Lai, Dong Liu, Anjie Hu, Lin Xu, Yongcan Chen

**Affiliations:** 1 School of Environment and Resources, Southwest University of Science and Technology, Mianyang 621010, China; 2 School of Civil Engineering and Architecture, Southwest University of Science and Technology, Mianyang 621010, China; 3 School of Economics and Management, Southwest University of Science and Technology, Mianyang 621010, China

**Keywords:** sludge, low temperature drying, COMSOL, numerical simulation, heat and mass transfer

## Abstract

Based on the sludge mass transfer flux model, this paper conducts a simulation study on the drying characteristics of sludge under low-temperature environment and compares it with the previous experimental results. It is found that when the sludge moisture content is low, the change of its drying curve is basically consistent with the experimental results, but there is a large error when the sludge moisture content is 0.4–0.6. In order to better simulate sludge drying characteristics, a model of cracking and shrinkage coefficients based on sludge moisture content is proposed, and the effective diffusion coefficient and mass transfer coefficient are modified. The maximum error between simulation and experiment is reduced to 23.78%. Based on this model, the sludge drying mechanism was studied. It was found that heat transfer and diffusion played a major role in the initial stage of sludge drying, and diffusion played a major role in sludge drying 30 min later.

## 1. Introduction

With the continuous improvement of people’s living standards, the sewage treatment capacity is increasing. In addition, sludge is the product of sewage treatment plant. Its treatment capacity is also increasing [1,2,3]. The 2015 Paris Agreement initiated a global commitment and effort to reduce CO2 emissions to lower the risks and impacts of climate change by limiting the increase in global average temperatures to well below 2 °C above pre-industrialized levels, aiming for 1.5 °C and to reach net-zero emissions in the second half of the century [4,5]. The reduction, hazard-free treatment, stabilization, and low carbonization of sludge treatment are important environmental issues at present [6]. Sludge drying is an important means of sludge reduction. It can reduce the mass and volume of sludge, kill pathogens, and reduce handling and transportation costs. At present, conventional drying methods include high-temperature thermal drying, vacuum drying, radiation drying, etc. However, there are many problems, such as large equipment investment, high drying energy consumption, high operation cost, serious pollution, and low safety. With the development of heat pump drying technology, low-temperature heat pump sludge drying technology has attracted more and more attention due to its energy-saving, environmental protection, safety and health, and good quality of drying products [7,8].

Sludge drying is a complex heat and moisture transfer process. In terms of heat and mass transfer research, Khalil UrRehman [9,10,11] noticed that in non-porous and porous media, t Nusselt number has a direct relationship with the change of Prandtl number for the mathematical model of heat transfer in Carreau fluid flow with physical effects and the corresponding solutions. At the same time, they also used group theory analysis, human intelligence, and other methods to study heat and mass transfer problems. In order to master the rules of sludge drying, many scholars have carried out research on the drying characteristics of sludge, in which the description of the drying process is the key [12]. Deng [13] and Vaxelaire [1] drew the drying curve through a large number of experiments, divided the drying process into four stages, and analyzed the moisture removed in different stages. However, Bennamoun [14] divided the drying process into three stages: the increasing speed stage, constant speed stage, and decreasing speed stage, and found that the decreasing speed drying stage accounted for a large proportion of the whole drying process. Zheng Q [15] studied the sludge drying characteristics under different air temperatures, wind speeds, and relative humidity. It was found that the effect of relative humidity on sludge drying characteristics at low temperatures could not be ignored, and the introduction of relative humidity into the drying model coefficient improved the prediction ability of the drying process. At present, although the research on sludge drying is very extensive, there is no widely applicable drying model. Even the drying model of the same kind of sludge under certain conditions is difficult to determine.

From the above studies, it can be seen that the researchers’ research on sludge drying is conducted through a large number of experiments, which are high in cost and low in efficiency. However, using computer software to simulate the drying process of porous media is a cheap, time-saving, and energy-saving method. In addition, it can also obtain detailed and accurate information about the sludge drying process.

For heat and mass transfer in porous media, Farahani et al. [16] studied the pore scale mechanism controlling heat transfer in partially saturated porous media under unfrozen and frozen conditions and developed a numerical model for predicting effective thermal conductivity (ETC). Jamaledine and Ray [17] studied several CFD analysis software to simulate the drying process of wet materials and found that software such as ANSYS FLUENT can be used to analyze fluid dynamics. However, FLUENT could not directly simulate the drying process of wet porous media (food, agricultural products, etc.) because FLUENT’s own control equations did not include energy source terms and water source terms [18,19,20]. Assari et al. [21] calculated the wheat drying process using CFD software based on the Euler two-fluid model, and the simulated values of temperature and humidity were in good agreement with the experimental values. Zare et al. [22] conducted a lot of research on the drying of porous media. However, their main research is carried out in a one-dimensional model, and in the drying of porous media, three-dimensional modeling and research are more consistent with the actual drying situation and more reliable. The numerical simulation of sludge drying, especially the simulation of the mass transfer mechanism, needs further research.

The numerical simulation of sludge drying characteristics can well predict the drying characteristics of sludge and analyze its heat and mass transfer mechanism from a microscopic perspective. In this paper, a three-dimensional model of the thin layer sludge drying model is established by numerical method, and its drying characteristics under low temperatures are studied. Compared with the previous experimental results [12], the convergence coefficient is proposed to optimize the model and the drying temperature. The influence of air humidity and other factors on sludge drying characteristics.

## 2. Numerical Simulation

### 2.1. Geometric Model

**Figure 1:**
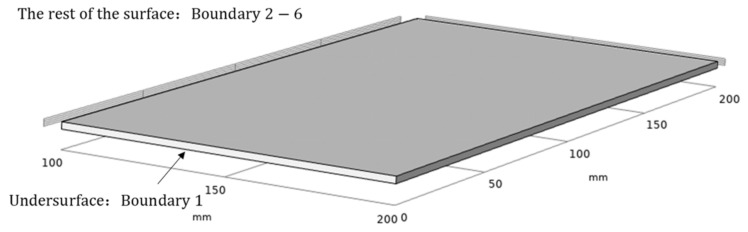
3D model of numerical simulation of sludge drying at low temperature.

Three-dimensional low-temperature drying model is built according to the size of the thin layer sludge, and the size of thin layer sludge is 200 mm × 200 mm × 2 mm cuboid. The middle position in the thickness direction is symmetrically simplified, so the calculation area of sludge low-temperature drying is determined as 200 mm × 200 mm × 1 mm cuboid, as shown in Figure 1, where the bottom of the model is defined as a symmetric boundary condition (boundary 1), and the surrounding boundary is a coupling boundary condition, while the other boundary interfaces are water evaporation heat flux boundaries (boundaries 2–6).

### 2.2. Control Equation of Heat and Mass Transfer in Sludge Drying

The sludge low-temperature drying process is a process of discharging water from sludge, mainly including heat and mass transfer inside sludge and heat and mass transfer between sludge surface moisture and environment [23,24]. The research purpose of the sludge drying process is to obtain the temperature field and humidity field inside the sludge, link them with the characteristic drying curve, reveal the drying law, and provide the basis for the selection of drying technology and drying process. The sludge low-temperature drying model is coupled with two transient interfaces describing temperature and moisture concentration, respectively. This model does not model the convective velocity field outside the sludge because the coefficient of convective heat and moisture transfer with the surrounding air has been given.

#### 2.2.1. Heat Transfer Control Equation

The heat transfer in sludge is calculated by the following energy conservation Equation (1) [25]:

(1) $$\rho C_{p}\frac{\partial T}{\partial t}+\rho C_{p}u\nabla T+\nabla \cdot q=Q+Q_{ted}$$

In Equation (1), $\rho C_{p}\frac{\partial T}{\partial t}$ represents the change of the unstable term of solid with time, $\rho C_{p}u\nabla T$ is the air convection heat transfer term in the sludge, and ∇·q is the heat transfer term in the sludge. *Q* is the internal heat source of sludge and $Q_{ted}$ is the external heat source of sludge. Wherein, the heat flow density q is expressed by Equation (2):

(2) q=−k∇T

where k is the thermal conductivity of sludge, W/(m·K).

#### 2.2.2. Mass Transfer Control Equation

The law of mass transfer describes the relationship between the flux of diffusing matter and the concentration gradient that generates mass transfer. Under the action of humidity gradient and temperature gradient, the moisture in the sludge will migrate to the outside in liquid form. The effective diffusion coefficient Deff [26,27] is introduced, and its mass transfer follows Fick’s second law. The equation of the transient concentration field in sludge is shown in Equation (3):

(3) $$\frac{\partial c_{j}}{\partial t}+\nabla \cdot J_{j}+u\nabla c_{j}=R_{j}$$

In the formula, $\frac{\partial c_{j}}{\partial t}$ is the change rate of the mass of water vapor in the unit volume of sludge with time, $\nabla \cdot J_{j}\;$ is the water diffusion mass flux caused by the concentration gradient of water vapor in the unit volume of sludge, and $u\nabla c_{j}$ is the mass flux of water vapor in the unit volume of sludge in three directions. $R_{j}$ is the external water vapor flux of sludge. Wherein the expression of water diffusion mass flux $J_{j}$ is Equation (4) [28]:

(4) $$J_{j}=-D_{eff}\nabla c_{j}$$

### 2.3. Boundary Condition

The evaporation of water on the outer boundary of sludge generates heat flux, and $\dot{I}$ is used in the boundary conditions of boundary 2–6 Term to represent this heat flux. Air convection increases the heat passing through boundaries 2–6. According to the previous assumption, another item is added to these boundary surfaces, which represents the heat flux transferred to the outside of the sludge due to water evaporation. To sum up, the boundary conditions of the heat transfer interface are expressed in Equations (5) and (6):

(5) $$n\cdot \left(-k\nabla T\right)=0\;\mathrm{Boundary}\;1$$

(6) $$n\cdot \left(-k\nabla T\right)=h_{T}\left(T_{air}-T\right)+\dot{I}\;\mathrm{Boundary}\;2–6$$

where $h_{T}$ is the heat transfer coefficient (W/(m^2^·K)) and $T_{air}$ is the air temperature in the drying oven.

Evaporative heat flux $\dot{I}$ As shown in Equation (7):

(7) $$\dot{I}=-I_{da}\cdot k\left(c_{0}-c\right)$$

where $I_{da}$ is the molar latent heat of evaporation, J/mol; $c_{0}\;$ is the concentration of air and water vapor in the drying oven, mol/m^3^.

The diffusion boundary conditions are shown in Equations (8) and (9):

(8) $$n\cdot \left(-D_{eff}\nabla c\right)=0\;\mathrm{Boundary}\;1$$

(9) $$n\cdot \left(D_{eff}\nabla c\right)=h_{m}\left(c_{0}-c\right)\;\mathrm{Boundary}\;2–6$$

where $D_{eff}$ is the water diffusion coefficient in the sludge, m^2^/s; $h_{m}$ is the convective mass transfer coefficient on the sludge surface, m/s; $c_{0}$ is the concentration of air and water vapor in the drying oven, mol/m^3^.

### 2.4. Determination of Physical Characteristic Parameters of Sludge

It is assumed that the shrinkage of sludge is ignored during the drying process. That is, the sludge volume remains unchanged. Measured according to the textbook Drainage Engineering, the maximum density of sludge is generally the highest when the water content is 65%, which is about 1220 kg/m^3^, the unit weight of 80% water content is 1140–1150 kg/m^3^, the 90% water content is 1010 kg/m^3^, and the 40% water content is 800–900 kg/m^3^, the water content of 30% is about 750 kg/m^3^, and it is concluded that the sludge density changes with the sludge water content. Therefore, sludge density is defined ρ the expression of is shown in Equation (10) [29]:

(10) $$\rho =1124-1036M^{2}+924$$

where M is the water content of the sludge.

The specific heat capacity at constant pressure is the internal energy absorbed or released when the unit mass object changes the unit temperature, and the specific heat capacity is the physical quantity representing the thermal property of the material, J/(kg·K). This model assumes that the specific heat capacity of sludge increases with the increase in temperature, and the specific heat capacity of sludge at constant pressure $C_{p}$ is shown in Equation (11) [30]:

(11) $$C_{p}=\left(2301+2.05T+0.24T^{2}+0.002T^{3}\right)$$

The thermal conductivity of sludge, *k*, W/(m·K) [31], is the heat transmitted through a unit area in unit time under the action of a unit temperature gradient, which increases with the increase in water concentration. Therefore, the expression for defining the thermal conductivity k of sludge is shown in Equation (12) [32]:

(12) $$k=0.194+0.436\left(\frac{cM_{H_{2}O}}{\rho }\right)$$

where, c is the water concentration of sludge, mol/m^3^; $M_{H_{2}O}$ is the molar mass of water, kg/mol.

The effective sludge diffusion coefficient Deff is considered to change with the change of sludge moisture content. The sixth-order polynomial is used to fit the rule of 12 groups of experimental data Deff–Mt, and the sixth-order polynomial function expression of Deff–Mt is obtained as shown in Equation (13) [33]:

(13) $$Deff=P_{1}Mt^{6}+P_{2}Mt^{5}+P_{3}tMt^{4}+P_{4}Mt^{3}+P_{5}Mt^{2}+P_{6}Mt+P_{7}$$

where Mt is the moisture content of sludge at time  t during drying (g/g).

In the drying process under the same working condition, the convective mass transfer coefficient $h_{m}$ [34] on the sludge surface is a constant value. The convective mass transfer coefficient hm of the sludge surface of 12 groups of experimental data is shown in Table 1.

### 2.5. Simulation Using COMSOL Multiphysics 5.5

Select the dilute material transfer module and solid heat transfer module in COMSOL Multiphysics 5.5. COMSOL Multiphysics is used to divide the mesh freely. The mesh is a tetrahedron mesh. The number of free meshes of the model is 91,925, and the volume is 40,000 m^3^. Set the calculation time and step size under different working conditions and enable the unsteady calculation method for calculation.

## 3. Analysis of Simulation Results

### 3.1. Analysis of Simulation and Experimental Results

**Figure 2:**
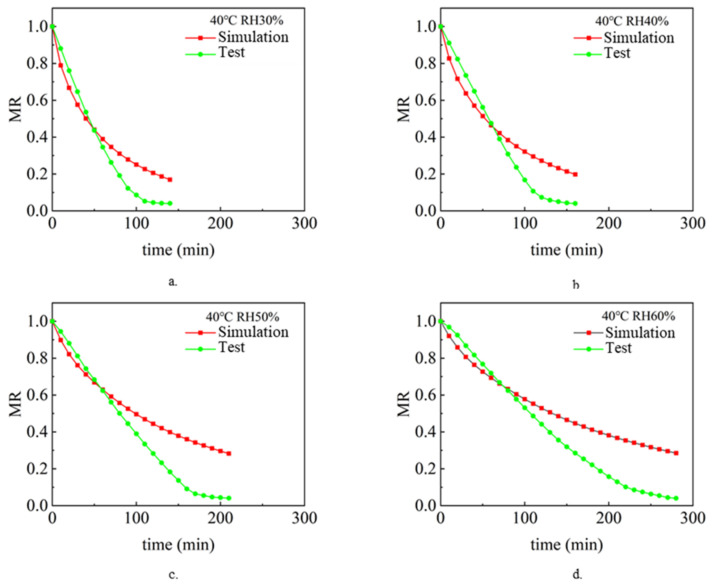
The simulated and experimental values of the change of the sludge moisture ratio MR with the drying time t when the air temperature is 40 °C: (**a**) RH 30%, (**b**) RH 40%, (**c**) RH 50%, (**d**) RH 60%.

**Figure 3:**
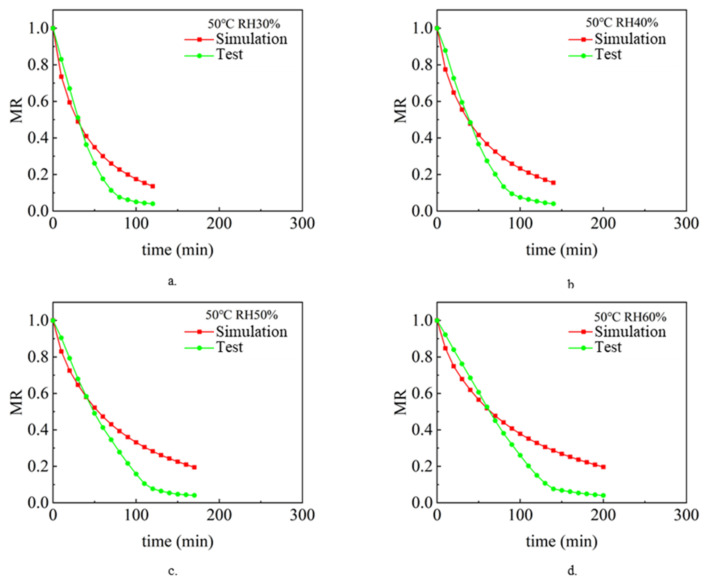
Changes of sludge moisture ratio MR with drying time t when the air temperature is 50 ℃: (**a**) RH 30%, (**b**) RH 40%, (**c**) RH 50%, (**d**) RH 60%.

**Figure 4:**
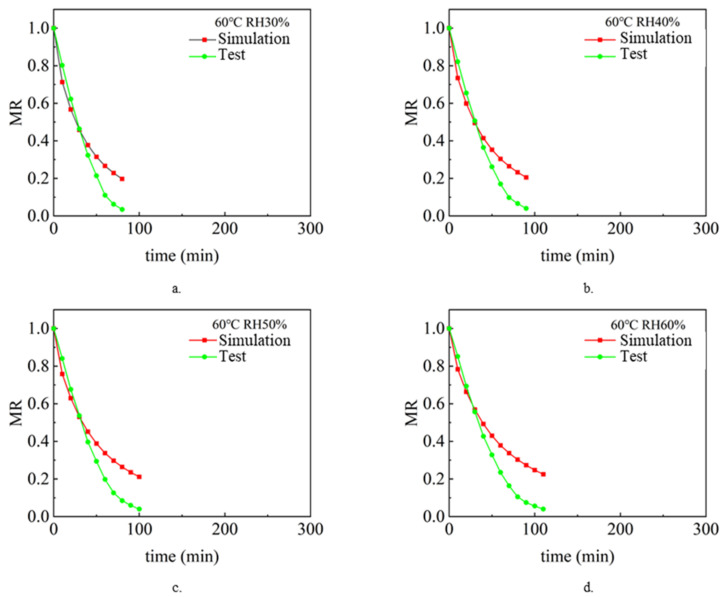
Variation of sludge moisture ratio MR with drying time t when the air temperature is 60 °C: (**a**) RH 30%, (**b**) RH 40%, (**c**) RH 50%, (**d**) RH 60%.

The drying process under different air temperature and air relative humidity conditions with a sludge thickness of 2 mm and inlet wind speed of 1.5 m/s was simulated and analyzed. Analyze and compare the simulation value and experimental value [9], and compare the change rule of sludge water ratio MR with drying time t so as to verify the accuracy of the model. Figure 2, Figure 3 and Figure 4 show the simulated and experimental values of sludge water ratio MR changing with drying time under different relative humidity conditions when the air temperature is 40 °C, 50 °C, and 60 °C, respectively. It can be seen from the diagram analysis. During the simulated drying process, the change in the drying curve was basically consistent with the experimental results before the sludge water ratio MR was 0.6. However, when the sludge water ratio MR is 0.4–0.6 during the drying process, it is found that the deviation is large. Specifically, the simulated drying rate is slower than the experimental drying rate. When the sludge moisture ratio MR is below 0.4 during drying, the error becomes larger and larger. This is because the internal structure of sludge changes during the actual drying process. When the sludge moisture ratio MR is 0.4–0.6 during the drying process, cracking [35] and shrinkage [36] occur in the sludge. During the process of hot air convection drying of sludge, with the diffusion and evaporation of water, the sludge will become smaller, shrink internally, and the structure of pores and pores between solid skeletons will change. When the sludge is dried to the stage where the sludge-water ratio MR is 0.6–0.4, the internal diffusion mechanism is mainly the loss of sludge-adsorbed water. At this time, the void caused by water loss can only be compensated by sludge shrinkage. Therefore, the sludge will crack and shrink. In the whole drying process under the same drying condition, the value of the effective moisture diffusion coefficient, considering the regular shrinkage of sludge, is smaller than that without sludge shrinkage. The appearance of cracking increases the contact area between sludge and hot air further increases the convective mass transfer coefficient, and also reduces the resistance of water diffusion. The water in sludge is easier to escape due to cracking. Sludge shrinkage and cracking have a continuous impact on the drying rate during sludge drying, and this impact will continue to increase.

It can be seen in Figure 2. During the first 70 min of the experimental and simulated values, the change rule of sludge water ratio MR with time is basically consistent, which indicates that the numerical simulation model of sludge drying is applicable, while the selection and calculation of physical parameters (sludge density, specific heat, thermal conductivity, diffusion coefficient and convection mass transfer coefficient on the sludge surface) are in line with the reality of the sludge drying process. However, after 70 min, the difference between the sludge water content of the experimental process and the simulated process became larger and larger, with the error ranging from 5.6% at the beginning to 110% at the end of drying. This shows that the shrinkage and cracking of sludge have a continuous impact on the drying rate during the sludge drying process, and this impact will continue to increase. Because the numerical model of sludge drying assumes that the volume of sludge does not change and shrinkage cracking is not considered in the calculation process, it is the main reason for large errors. The phenomena reflected in Figure 3 and Figure 4 are basically consistent with the above.

### 3.2. Model Modification

According to the above analysis, cracking during sludge drying will increase the convective mass transfer coefficient of the sludge surface, while sludge shrinkage will reduce the effective diffusion coefficient of sludge. Therefore, the cracking phenomenon and shrinkage phenomenon are described by modifying the physical parameters. Therefore, when defining the sludge surface convection mass transfer coefficient, hm, and the effective sludge diffusion coefficient Deff, the sludge cracking coefficient is added α. And sludge shrinkage coefficient β. To control the convective mass transfer coefficient hm on the sludge surface and the effective diffusion coefficient Deff of sludge so that the model calculation can reflect the cracking and shrinkage phenomena generated during the sludge drying process. Among a>1, 0<β<1.

(1) Modification of convective mass transfer coefficient hm on sludge surface in the model

In this experiment, when the sludge water ratio is 0.6, the corresponding sludge dry basis moisture content is 1.073. As the sludge shrinks, its influence on the drying rate becomes greater and greater, so the sludge cracking coefficient is defined α The sludge shrinkage coefficient at the beginning of shrinkage is $\alpha_{0}$. Sludge cracking coefficient α the expression of is shown in Equation (14):

(14) $$\alpha =\alpha_{0}\exp\left(aM_{t}\right)+b$$

where a and b are parameters, and the values of a and b are different under different working conditions. 

The modified definition of the effective sludge diffusion coefficient $\;h_{m}{}^{\prime }$ is shown in Equations (15) and (16):

(15) $$h_{m}{}^{\prime }=h_{m}\;Mt\geq 1.073$$

(16) $$h_{m}{}^{\prime }=\alpha h_{m}\;Mt<1.073$$

(2) Modification of sludge effective diffusion coefficient Deff in the model

As the effect of sludge shrinkage on drying rate becomes greater and greater, the sludge shrinkage coefficient is defined β. The sludge shrinkage coefficient at the beginning of shrinkage is $\beta_{0}$. Sludge shrinkage coefficient β. The expression of is shown in Equation (17):

(17) $$\beta =c\beta_{0}+dM_{t}$$

where, c and d are parameters, and the values of *c* and *d* are different under different working conditions.

The modified definition of the effective sludge diffusion coefficient $D_{eff}{}^{\prime }$ is shown in Equations (18) and (19):

(18) $$D_{eff}{}^{\prime }=P_{1}Mt^{6}+P_{2}Mt^{5}+P_{3}tMt^{4}+P_{4}Mt^{3}+P_{5}Mt^{2}+P_{6}Mt+P_{7}Mt\geq 1.073$$

(19) $$D_{eff}{}^{\prime }=\beta \left(P_{1}Mt^{6}+P_{2}Mt^{5}+P_{3}tMt^{4}+P_{4}Mt^{3}+P_{5}Mt^{2}+P_{6}Mt+P_{7}\right)Mt<1.073$$

Coefficient under different working conditions α and coefficient β. The determination is shown in Table 2.

### 3.3. Analysis of Simulation and Experimental Results after Model Modification

**Figure 5:**
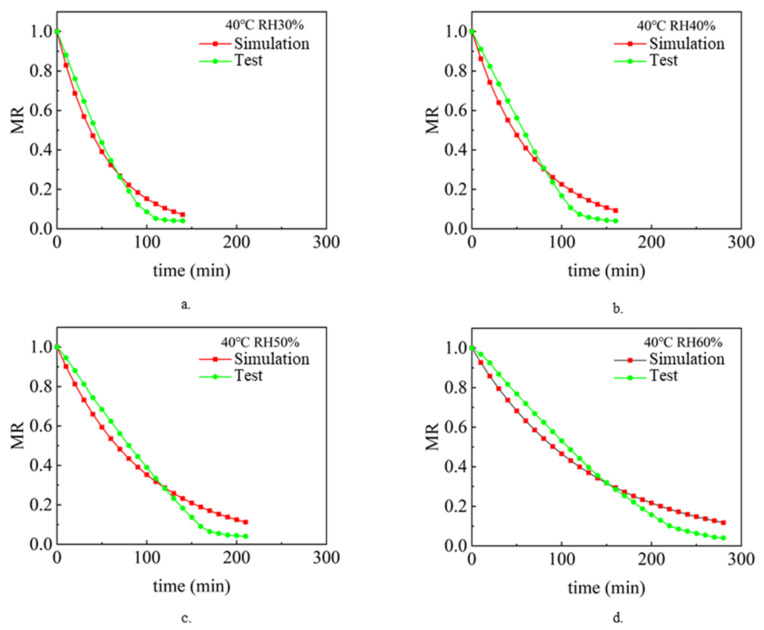
The simulated and experimental values of the change of the sludge moisture ratio MR with the drying time t when the air temperature is 40 °C after considering the shrinkage effect and the crack phenomenon during the sludge drying process: (**a**) RH 30%, (**b**) RH 40%, (**c**) RH 50%, (**d**) RH 60%.

**Figure 6:**
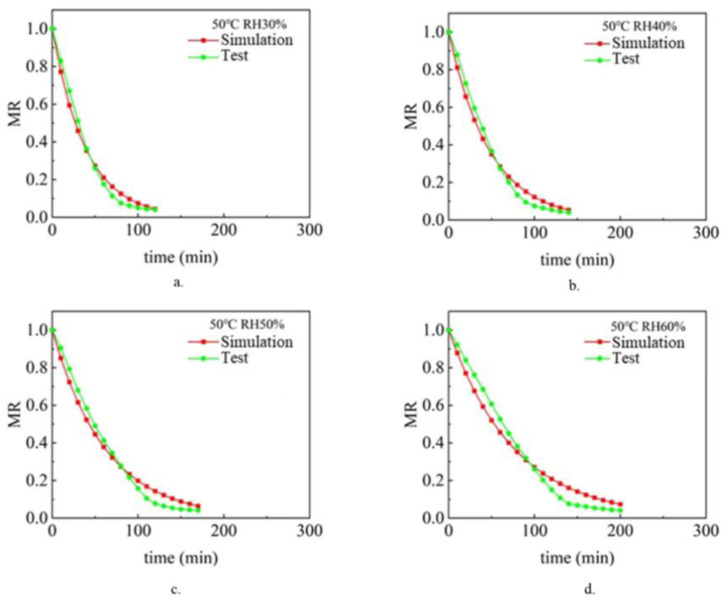
The simulated and experimental values of the change of the sludge moisture ratio MR with the drying time t when the air temperature is 50 °C after considering the shrinkage effect and the crack phenomenon in the sludge drying process: (**a**) RH 30%, (**b**) RH 40%, (**c**) RH 50%, (**d**) RH 60%.

**Figure 7:**
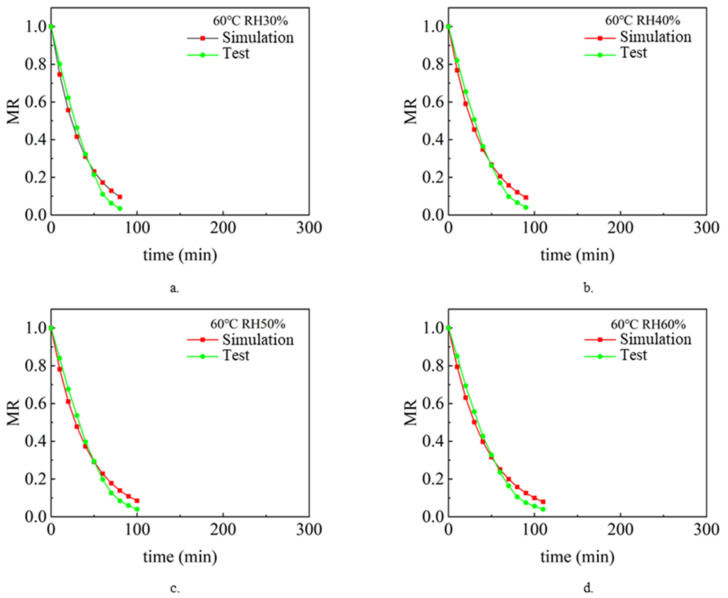
The simulated and experimental values of the change of the sludge moisture ratio MR with the drying time t when the air temperature is 60 °C after considering the shrinkage and cracks during the sludge drying process: (**a**) RH 30%, (**b**) RH 40%, (**c**) RH 50%, (**d**) RH 60%.

Figure 5, Figure 6 and Figure 7 show the simulated and experimental values of sludge water ratio MR changing with drying time t under different air relative humidity conditions at 40 °C, 50 °C, and 60 °C after considering the shrinkage and cracking phenomena during sludge drying. According to the results in the figure, after modifying the effective diffusion coefficient Deff of sludge in the model and the convective mass transfer coefficient hm of sludge surface in the model, the experimental and simulated values of sludge water ratio change with drying time are basically consistent. Table 3 shows the error between the simulated value and the experimental value. It can be seen from the table that the error between the simulated value and the experimental value is within 23.78%. The simulated drying process enters the drying equilibrium state earlier than the actual drying process. This is due to experimental errors. During the experiment, due to the rapid drying of the sludge surface, part of the sludge is transformed into powder dust. With the drying process, this powder dust-like sludge is blown away by the flowing air, resulting in a lower sludge quality during the measurement, so the measured sludge drying rate is faster than the actual drying rate.

### 3.4. Average Temperature Analysis

**Figure 8:**
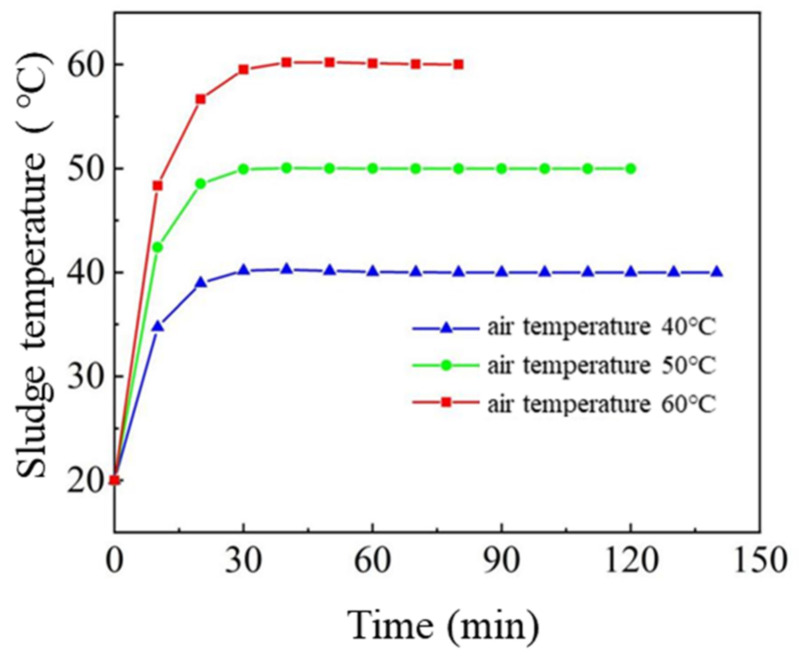
The average temperature rise curve inside the sludge at different air temperatures under the condition of 30% relative air humidity.

Figure 8 shows the average temperature rise curve inside the sludge at different air temperatures under the condition of 30% relative air humidity. The results showed that the average temperature of sludge increased to an equilibrium temperature about 30 min after drying. In the initial stage of drying, the internal temperature of sludge quickly reaches the temperature of drying air, which indicates that the internal heat transfer resistance of sludge is very weak under specific drying conditions. This also shows that the water transfer mechanism in the sludge drying process is controlled by heat transfer and diffusion mechanisms 30 min before the drying time, while it is mainly controlled by diffusion mechanisms 30 min after the drying time.

### 3.5. Isothermal Surface Analysis of Sludge

**Figure 9:**
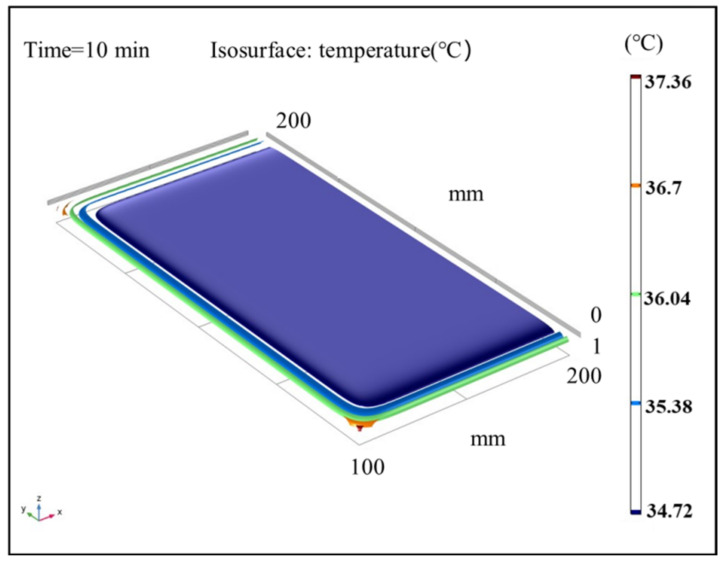
Isothermal surface distribution cloud map of average sludge temperature at 10 min.

**Figure 10:**
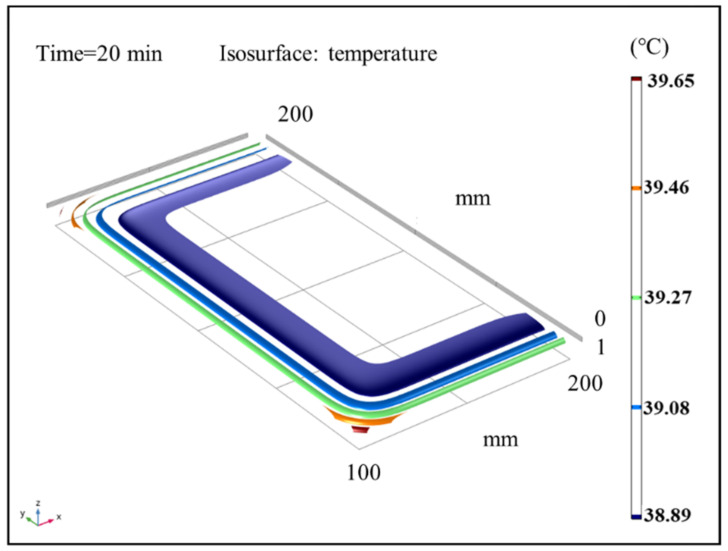
Isothermal surface distribution cloud map of average sludge temperature at 20 min.

**Figure 11:**
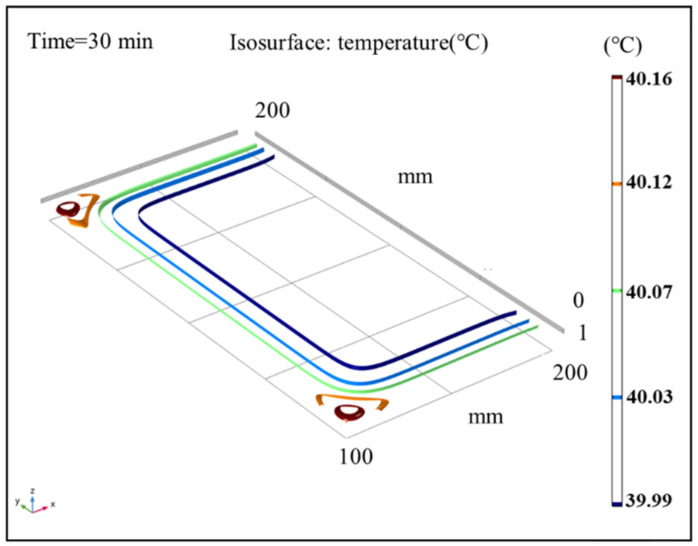
Cloud map of the isothermal surface distribution of average sludge temperature at 30 min.

The results of the temperature field in sludge under different time steps were analyzed under the conditions of an air temperature of 40 °C and air relative humidity of 60%. Figure 9, Figure 10 and Figure 11 show the sludge drying process to 10 min, 20 min, and 30 min, respectively. The results show that the temperature distribution is a gradient from high outside to low inside. The surface and boundary temperature of sludge will quickly approach the set air temperature after drying. In the heat flow transfer process, the temperature in the upper left and right corners of the sludge transfers fastest and has the highest temperature. During the period of 10 min to 20 min, the change speed of the temperature field decreased; After 20 min, the temperature of the sludge changed slowly. Finally, the temperature field inside the sludge is completely stable at 30 min, and the temperature of the whole sludge reaches the air temperature, with a temperature gradient close to 0.

### 3.6. Analysis of Water Transfer in Sludge

**Figure 12:**
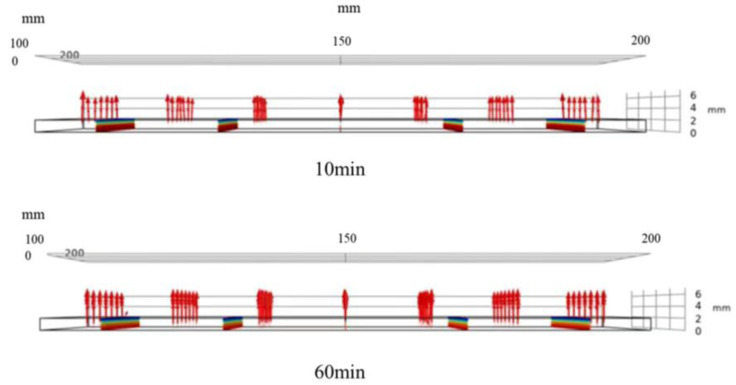
Under the condition of an air temperature of 50 °C and relative air humidity of 50%, the diffusion direction of water inside the sludge at different temperatures of 10 min and 60 min.

**Figure 13:**
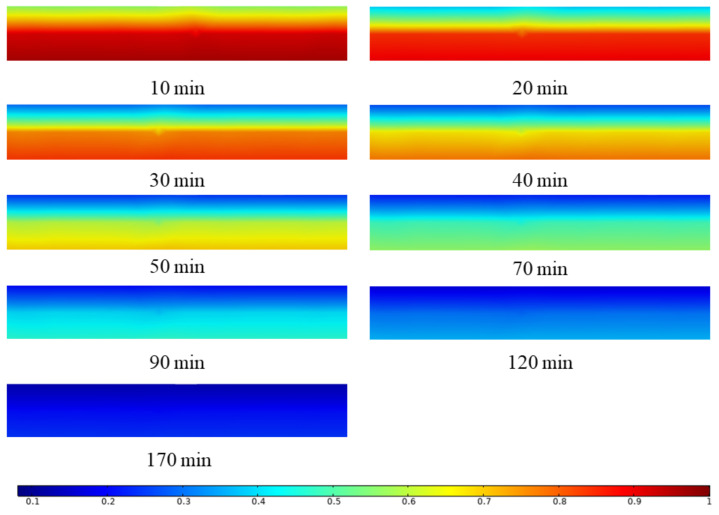
MR distribution cloud map of sludge moisture ratio in a section of the sludge center at different times under the conditions of air temperature 50 °C and air relative humidity 50%.

The change of sludge water content is described by calculating the sludge water ratio MR of different time steps. Figure 12 shows the diffusion direction of water in sludge at 10 min and 60 min under the conditions of an air temperature of 50 °C and air relative humidity of 50%. According to the above analysis, the average temperature of the sludge reached the equilibrium temperature at 30 min, so the diffusion of water inside the sludge at two stages before and after 30 min was observed. It can be seen from the figure that at 10 min and 60 min, the diffusion of water in sludge is one-dimensional diffusion in the -direction. Since sludge drying can be regarded as one-dimensional diffusion, a section of sludge center ZX is taken for analysis. Figure 13 shows the MR distribution cloud diagram of sludge water ratio in one section of the sludge center at different times under the working conditions of an air temperature of 50 °C and air relative humidity of 50%. It can be seen from the figure that when the sludge is dried for 10 min, the water ratio MR of the sludge surface has reached the range of 0.6–0.7, while the water ratio MR of the sludge interior is still in the range of 0.9–1. The reason is that 10 min before drying belongs to the drying acceleration stage, and the main mechanism of water migration in this stage is the evaporation of free water on the sludge surface. When the sludge is dried for 20 min, it can be seen that the sludge surface water ratio MR rapidly drops to the range of 0.3–0.4, while the sludge internal water ratio MR starts to change slowly, reaching the level of 0.8–0.9, which can be seen that the diffusion in the sludge starts at this stage. When the sludge is dried for 30 min, the sludge surface water ratio MR has nearly reached the state of drying balance, and the water concentration inside and outside the sludge has formed a significant gradient. This is because the free water on the sludge surface has evaporated completely, and the main mechanism of water transfer at this stage is the diffusion inside the sludge. During the 40–120 min stage of sludge drying, the sludge surface water ratio (MR) is basically unchanged, maintained at about 0.1, and the water in the sludge continues to diffuse, with the diffusion rate gradually decreasing. When the sludge is dried for 120 min, the internal diffusion rate of the sludge is greatly reduced. The reason is that at this time, the water content of the sludge quickly reaches a stable water content, the free water content in the sludge is already very low, and the combined water of the sludge can no longer be further dehydrated due to the effect of sewage temperature and concentration difference. Therefore, the water content in the sludge changes slowly from 120 min to 170 min until the sludge reaches the drying equilibrium state. To sum up, during the period of 10 min–30 min, the distribution of water content inside the sludge is that the water content on the sludge surface decreases rapidly, while the water content inside the sludge changes slowly; During 30 min–40 min, the gradient of sludge moisture content tends to be stable; During the period of 40 min–120 min, the moisture content gradient of sludge on dry basis reached a uniform state; During the period of 120 min to 170 min, the moisture content of sludge on dry basis changes slowly until the sludge is dried into a balanced state.

### 3.7. Evaluation of Numerical Model for Sludge Low-Temperature Drying

The simulated sludge low-temperature drying numerical model considering the phenomena of sludge cracking and sludge shrinkage in the sludge drying process was used for simulation. The change in sludge moisture content obtained through comparative analysis was basically consistent with the experimental drying process, reflecting the three stages of sludge drying and accurately describing the distribution of sludge internal moisture content at different times of sludge drying. It can be used in practical projects. In addition, the numerical simulation of sludge low-temperature drying can also reflect the change of internal sludge temperature, which provides theoretical support for the research of sludge drying.

## 4. Conclusions

In this paper, the heat and mass transfer in the low-temperature sludge drying process was numerically simulated. The conclusions are as follows:

1. Comparing the simulation results with the experimental results, it can be observed that the change in the drying curve is basically consistent with the experimental results before the sludge water ratio MR is 0.6 in the simulated drying process. However, when the sludge moisture ratio MR is 0.4–0.6 during drying, a large deviation is found. The reason is that the numerical simulation did not consider the phenomenon of sludge cracking and sludge shrinkage during the sludge drying process.
2. Based on the phenomenon of sludge cracking and shrinkage, the sludge cracking coefficient is defined α, and the sludge shrinkage coefficient β, the effective diffusion coefficient, and the mass transfer coefficient were corrected, and the error between simulation results and experimental results was within 23.78%.
3. Through the analysis of the average temperature change of sludge, it is concluded that the average temperature inside the sludge rises to the equilibrium temperature about 30 min after the drying. The water transfer mechanism in the sludge drying process is controlled by heat transfer and diffusion before 30 min of drying time, while it is mainly controlled by the diffusion mechanism after 30 min of drying time. Through the analysis of sludge’s internal isothermal surface, it is concluded that the surface and boundary temperature of sludge quickly approach the set air temperature after the beginning of drying. Through the analysis of the sludge concentration change diagram, it is concluded that the water diffusion in the sludge is one-dimensional diffusion in the z-direction, and the sludge drying characteristics can be observed, which is consistent with the thin-layer sludge dynamics theory.

## Figures and Tables

**Table 1:** The estimated value of convective mass transfer coefficient on sludge surface under different air temperatures and relative humidity.

Temp (°C)	Relative Humidity (%)	$h_{m}\;(m/s*10{-}^{7})$
40	30	3.89
	40	3.01
	50	1.56
	60	1.17
50	30	5.56
	40	4.42
	50	3.05
	60	2.70
60	30	6.50
	40	6.09
	50	5.20
	60	4.57

**Table 2:** Coefficient α and coefficient β under different working conditions.

Temp (°C)	Relative Humidity (%)	Parameters	$a_{0}$	$\beta_{0}$
40	30	a = −1.1423, b = 1.2644 c = 0.9012, d = 0.1123	1.2000	0.9000
	40	a = −1.3523, b = 1.2325 c = 0.8862, d = 0.1089	1.2000	0.9000
	50	a = −1.4011, b = 1.2089 c = 0.8726, d = 0.1012	1.2000	0.9000
	60	a = −1.4628, b = 1.1843 c = 0.8012, d = 0.0989	1.2000	0.9000
50	30	a = −1.1177, b = 1.3049 c = 0.8912, d = 0.1045	1.2000	0.9000
	40	a = −1.2265, b = 1.2410 c = 0.864, d = 0.1012	1.2000	0.9000
	50	a = −1.2847, b = 1.1654 c = 0.8499, d = 0.1001	1.2000	0.9000
	60	a = −1.3014, b = 1.1543 c = 0.8162, d = 0.1013	1.2000	0.9000
60	30	a = −1.0875, b = 1.3785 c = 0.874, d = 0.1011	1.2000	0.9000
	40	a = −1.1236, b = 1.3461 c = 0.864, d = 0.1036	1.2000	0.9000
	50	a = −1.2548, b = 1.2977 c = 0.858, d = 0.1044	1.2000	0.9000
	60	a = −1.2998, b = 1.2413 c = 0.826, d = 0.0984	1.2000	0.9000

**Table 3:** Error table between simulated and experimental values.

Temp (°C)	Relative Humidity (%)	Error (%)
40	30	23.78
	40	21.05
	50	18.50
	60	19.33
50	30	20.75
	40	21.17
	50	23.63
	60	15.60
60	30	21.39
	40	22.11
	50	20.63
	60	21.85

## Data Availability

Not applicable.

## Nomenclature

ρ	sludge density	k	thermal conductivity of sludge
$C_{p}$	Specific heat capacity of sludge at constant pressure	$c_{j}$	quality of water vapor
T	temperature	$J_{j}$	expression of water diffusion mass flux
t	time	$R_{j}$	external water vapor flux of sludge
u	air flow velocity	$D_{eff}$	effective moisture diffusivity
q	heat flux	$h_{T}$	heat transfer coefficient
Q	internal heat source of sludge	$T_{air}$	air temperature in the drying oven
$Q_{ted}$	the external heat source of sludge	$\dot{I}$	evaporative heat flux
$I_{da}$	molar latent heat of evaporation	$c_{0}$	concentration of air and water vapor in the drying oven
c	sludge moisture concentration	$h_{m}$	convective mass transfer coefficient on the sludge surface
$M_{H_{2}O}$	molar mass of water	M	water content of sludge
Mt	moisture content of sludge on dry basis at any drying time	MR	dimensionless sludge moisture content ratio
α	sludge cracking coefficient	β	sludge shrinkage coefficient

## References

[1] Vaxelaire J., Cézac P. (2004). Moisture distribution in activated sludges: A review. Water Res..

[2] Zhang Q.H., Yang W.N., Ngo H.H., Guo W.S., Jin P.K., Dzakpasu M., Yang S.J., Wang Q., Wang X.C., Ao D. (2016). Current status of urban wastewater treatment plants in China. Environ. Int..

[3] Tunçal T., Uslu O. (2014). A Review of Dehydration of Various Industrial Sludges. Dry. Technol. Int. J..

[4] Hassanpouryouzband A., Joonaki E., Edlmann K., Haszeldine R.S. (2021). Offshore Geological Storage of Hydrogen: Is This Our Best Option to Achieve Net-Zero?. ACS Energy Lett..

[5] Hassanpouryouzband A., Joonaki E., Edlmann K., Haszeldine R.S. (2022). Geological Hydrogen Storage: Geochemical Reactivity of Hydrogen with Sandstone Reservoirs. ACS Energy Lett..

[6] Bogler A., Packman A., Furman A., Gross A., Kushmaro A., Ronen A., Dagot C., Hill C., Vaizel-Ohayon D., Morgenroth E. (2020). Rethinking wastewater risks and monitoring in light of the COVID-19 pandemic. Nat. Sustain..

[7] Zhang J., Zhang H.H., He Y.L., Tao W.Q. (2016). A comprehensive review on advances and applications of industrial heat pumps based on the practices in China. Appl. Energy.

[8] Zhang H., Su L., Lv T., Dong K. (2019). Coupling Heat Pump and Vacuum Drying Technology for Urban Sludge Processing. Energy Procedia.

[9] Rehman K.U., Shatanawi W., Abodayeh K. (2022). A group theoretic analysis on heat transfer in MHD thermally slip Carreau fluid subject to multiple flow regimes (MFRs). Case Stud. Therm. Eng..

[10] Rehman K.U., Shatanawi W., Abodayeh K., Shatnawi T.A. (2022). A Group Theoretic Analysis of Mutual Interactions of Heat and Mass Transfer in a Thermally Slip Semi-Infinite Domain. Appl. Sci..

[11] Rehman K.U., Shatanawi W., Çolak A.B. (2022). Thermal analysis of flowing stream in partially heated double forward-facing step by using artificial neural network. Case Stud. Therm. Eng..

[12] Wang Z., Xu L., Liu D., Zhang Q., Hu A., Wang R., Chen Y. (2021). Effects of Air Temperature and Humidity on the Kinetics of Sludge Drying at Low Temperatures. Energies.

[13] Deng W.Y., Yan J.H., Li X.D., Wang F., Lu S.Y., Chi Y., Cen K.F. (2009). Measurement and simulation of the contact drying of sewage sludge in a Nara-type paddle dryer. Chem. Eng. Sci..

[14] Bennamoun L., Fraikin L., Léonard A. (2014). Modeling and Simulation of Heat and Mass Transfer During Convective Drying of Wastewater Sludge with Introduction of Shrinkage Phenomena. Dry. Technol..

[15] Lewis W.K. (1921). Journal of industrial and engineering chemistry. Tournal Am. Ceram. Soc..

[16] Vasheghani Farahani M., Hassanpouryouzband A., Yang J., Tohidi B. (2020). Heat Transfer in Unfrozen and Frozen Porous Media: Experimental Measurement and Pore-Scale Modeling. Water Resour. Res..

[17] Jamaleddine T.J., Ray M.B. (2010). Application of Computational Fluid Dynamics for Simulation of Drying Processes: A Review. Dry. Technol..

[18] Zarea M.R.D. (2012). CFD Modeling of Microwave-Assisted Fluidized Bed Drying of Moist Particles Using Two-Fluid Model. Dry. Technol..

[19] Hacıhafızoğlu O., Cihan A., Kahveci K. (2008). Mathematical modelling of drying of thin layer rough rice. Food Bioprod. Process..

[20] Zare D., Ranjbaran M. (2012). Simulation and Validation of Microwave-Assisted Fluidized Bed Drying of Soybeans. Dry. Technol..

[21] Assari M.R., Tabrizi H.B., Najafpour E. (2013). Energy and exergy analysis of fluidized bed dryer based on two-fluid modeling. Int. J. Therm. Sci..

[22] Ranjbaran M., Zare D. (2013). Simulation of energetic- and exergetic performance of microwave-assisted fluidized bed drying of soybeans. Energy.

[23] Ma D., Li A., Zhang L., Wang D., Ji G. (2021). Mechanical compression assisted conductive drying of thin-film dewatered sewage sludge: Process performance, heat and mass transfer behavior–ScienceDirect. Waste Manag..

[24] Krawczyk P., Badyda K. (2011). Two-dimensional CFD modeling of the heat and mass transfer process during sewage sludge drying in a solar dryer. Arch. Thermodyn..

[25] Thorpe G.R. (2008). The application of computational fluid dynamics codes to simulate heat and moisture transfer in stored grains. J. Stored Prod. Res..

[26] Zhou J., Zhang R., Wang X., Chen S., Luo A., Niu D., Chai X., Zhao Y. (2016). NaHCO_3_-enhanced sewage sludge thin-layer drying: Drying characteristics and kinetics. Dry. Technol..

[27] Zhang X.Y., Chen M.Q., Huang Y.W., Xue F. (2016). Isothermal hot air drying behavior of municipal sewage sludge briquettes coupled with lignite additive. Fuel.

[28] Liang J., Tan J., Jiang B. (2022). Thermal and humid environment of rammed-earth dwellings in Northwest Sichuan. Indoor Built Environ..

[29] Zhang Z. (2000). Drainage Works Volume II.

[30] Chen H., Marks B.P., Murphy R.Y. (1999). Modeling coupled heat and mass transfer for convection cooking of chicken patties. J. Food Eng..

[31] Vesilind P.A., Martel C.J. (1989). Thermal conductivity of sludges. Water Res..

[32] Jingyu F. (2011). Experimental Study on Sludge Drying Mechanism.

[33] Mou X., Chen Z. (2021). Experimental study on the effect of sludge thickness on the characteristics of ultrasound-assisted hot air convective drying municipal sewage sludge. Dry. Technol..

[34] Wu H., Wang S., Kong H., He W., Xia M. (2007). Determination of bulk mass transfer coefficient of biosorption on sludge granule based on liquid membrane mass transfer mechanism. Bioresour. Technol..

[35] Wang P., Tang C.-S., Wang D., Du Y. (2016). Experimental investigation on dry evaporation and cracking behavior of fiber reinforced municipal sludge. J. Southeast Univ. (Nat. Sci. Ed.).

[36] Li J., Plougonven E., Fraikin L., Salmon T., Toye D., Léonard A. (2015). Image analysis of X-ray tomograms of sludge during convective drying in a pilot-scale fixed bed. Chem. Eng. Sci..

